# Heterogeneity in Community Size Effects: Exploring Variations in the Production of National Hockey League Draftees Between Canadian Cities

**DOI:** 10.3389/fpsyg.2018.02746

**Published:** 2019-01-14

**Authors:** Lou Farah, Jörg Schorer, Joseph Baker, Nick Wattie

**Affiliations:** ^1^School of Kinesiology and Health Science, York University, Toronto, ON, Canada; ^2^Institute of Sport Science, Carl von Ossietzky University of Oldenburg, Oldenburg, Germany; ^3^Faculty of Health Sciences, University of Ontario Institute of Technology, Oshawa, ON, Canada

**Keywords:** community size effect, athlete development, sport, expertise, city size, elite athletes

## Abstract

Previous research has explored ‘community size effects’ in a multitude of sporting and regional contexts and has shown that athletes are more likely to originate from small-medium population size categories, and less likely to originate from very small or large ones. However, it is not clear whether the production of athletes is homogenous within population size categories. Place of birth data were collected for all Canadian born hockey players drafted into the National Hockey League (NHL) from 2000–2014 from British Columbia (*N* = 192), Alberta (*N* = 218), Saskatchewan and Manitoba (*N* = 216), Ontario (*N* = 561), Quebec (*N* = 241), and the Atlantic Provinces (*N* = 74). To explore variations in the production of draftees within population size categories, proportions of productive cities, population mean (μ), population standard deviation (σ), as well as minimum/maximum values of the number of draftees were calculated for the different categories (<2,500; 2,500–4,999; 5,000–9,999; 10,000–29,999; 30,000–99,999; 100,000–249,999; 250,000–499,999; 500,000–999,999; >1,000,000). In addition, the number of draftees produced per 1,000 residents (i.e., yield) was calculated for each city within all categories. Results showed substantial intra-categorical variability in NHL talent development; moreover, heterogeneity in draftee production existed in various degrees across provincial regions of Canada. Intra-categorical variability suggests that a single homogenous community size effect may not exist for Canadian NHL draftees, and that future research may benefit from exploring other environmental constraints on athlete development such as income, population density, and proximity to local sport clubs.

## Introduction

Elite athletes reflect the end result of a long and complex developmental process (Ericsson et al., 1993; Baker et al., 2017). Consequently, unique and diverse factors make it challenging to predict future elite performance (Baker and Horton, 2004; Davids and Baker, 2007). One factor that has emerged over the past few decades is the ‘community size effect’ (Côté et al., 2006), referring to the influence of the population size of one’s place of birth on the likelihood of becoming an elite athlete. This concept was first introduced by Curtis and Birch (1987) when they compared the distribution of North American Olympic and professional ice hockey players across different population size categories to that of the North American general population. They observed that hockey players at these levels were overrepresented in small to medium sized cities, and underrepresented in regions of less than 1,000 and greater than 500,000 residents.

Community size effects have since been observed in multiple sports across numerous countries. For example, it has been shown that the optimal population size to develop American baseball, basketball, and golf players is between 50,000–99,999 residents (Côté et al., 2006). In Germany, Olympians are overrepresented in areas of 30,000–99,999 people (Baker et al., 2009). As for the sport of ice-hockey (hereafter simply referred to as hockey), World Junior (WJR) hockey players are overrepresented in populations of <10,000 in Sweden, 10,000–30,000 in Finland, and 100,000–500,000 in North America (Bruner et al., 2011). While several studies have observed community size effects, results have also indicated variation in the existence of the effect. For instance, Lidor et al. (2010) did not observe an effect in Division 1 male basketball players in Israel despite finding such effects in soccer, handball, and volleyball. Similarly, Lidor et al. (2014) observed this effect in Israeli females competing in Division 1 basketball, handball, and volleyball, but not in soccer. Along with inconsistencies in the existence of community size effects in certain sports or regions, some variations in this effect have also been observed between examined countries (e.g., Baker et al., 2009; Bruner et al., 2011), demonstrating incongruity in the ideal population size to develop certain athletes.

Rooney (1969) first highlighted an assumption that might have important implications for the generalizability of community size effects and for the variability of trends between studies. Rooney proposed that exploring the production of players between regions (i.e., states) only, but not within them, assumed uniformity of talent production across all cities *within* states, which may be misleading. For example, not all cities within the state of California might produce players at a similar capacity. Moreover, he noted that while the most populated states did generally produce more college football players than other states, the correlation between population size and talent production cannot be generalized across *all* regions of the same population size. To emphasize this point, Rooney noted that the state of New York had produced less than 50% of the number of players California had produced, despite their population sizes being virtually equal. Indeed, recent research has reinforced the importance of considering variation within community size effects (Wattie et al., 2018), noting that previous research on Canadian NHL athletes had only considered the aggregated general and athlete population distributions at a national level. The authors observed variation in general population distribution between provinces, as well as significant variation in the production of athletes across population size categories between provinces. Significant inter-provincial variation shown by Wattie et al. (2018) suggests that data aggregation at a national level masks substantial heterogeneity in NHL talent development between Canadian provinces. In the same sense, aggregating data on a provincial level may mask similar, if not more substantial, variation that exists between cities themselves; more specifically, cities of similar population sizes.

To date, studies of the community size effect have only compared the distribution of players between population size categories but not within them, which presents two notable limitations. First, aggregating the number of players produced by different cities in each population size category suggests a uniformity in player production across all cities within these categories, and thus may lead to overlooking important variation in athlete production. For example, Baker et al. (2014) highlighted the importance of studying the community size effect within population size categories by noting that both Vancouver (population size of 410,000) and Edmonton (population size of 460,000) fell into the same population size category (i.e., 250,000–499,999), yet Edmonton produced 26 National Hockey League (NHL) draftees while Vancouver had only managed to produce nine NHL draftees during the same period. This suggests cities of similar population sizes do not necessarily have equivalent athlete development environments, and that the generalizability of this effect may not apply to all regions within each population size category.

In addition, studies in this area have only included cities that have produced players, and generalized their findings across sporting contexts and countries without taking unproductive cities, which can form the vast majority of some regions, into account. Similar to data aggregation discussed above, the inclusion of only productive cities may mask even more variability in athletic talent production, therefore raising more questions regarding the validity and generalizability of this effect. Given variations in community size effects observed by Wattie et al. (2018) between provinces and the anecdotal observation of intra-population category variation by Baker et al. (2014), it may be useful to compare the production of athletes between cities of similar population sizes without excluding unproductive cities. The significance of testing the assumptions that community size effects are consistent within population size categories lies not only in its application toward the development of past Canadian hockey players, but also for how community size effects are discussed in contemporary contexts. Findings from this study may also open more avenues for future research to explore differences in developmental characteristics between cities, which can increase our knowledge and understanding of athlete development and its influences.

Therefore, the purpose of this research was to examine possible variation of community size effects in Canadian born hockey players drafted into the NHL between 2000–2014 by (i) determining the percentage of cities within each population size category, in every provincial region, that had managed to produce any NHL draftees, and (ii) examining the variation in the number of draftees produced between productive cities in each population size category.

## Materials and Methods

### Sample and Variables

Place of birth data were collected from the NHL website ^1^ for all Canadian born hockey players drafted into the NHL from 2000–2014 (*N* = 1502). In order to compare community size effects across different regions of Canada, draftees were categorized based on the provinces from which they originated. However, given the relatively small number of draftees produced by New Brunswick, Newfoundland and Labrador, Nova Scotia, and Prince Edward Island (*N* = 74), and their geographical proximity to each other, these provinces were grouped and coded as the “Atlantic Provinces.” Similarly, Saskatchewan and Manitoba are neighboring provinces and had relatively low individual productions of 124 and 92 draftees, respectively. Consequently, these two provinces were coded as “Saskatchewan and Manitoba.” As a result, the following provincial regions were used in this study: British Columbia (*N* = 192), Alberta (*N* = 218), Saskatchewan and Manitoba (*N* = 216), Ontario (*N* = 561), Quebec (*N* = 241), and the Atlantic provinces (*N* = 74).^2^

The population size of athletes’ place of birth was obtained from publically available Canadian census data from Statistics Canada for the years of 1991 and 1996. These particular census years were chosen as they most closely resemble the time during which the athletes’ participation took place (i.e., 5–15 years of age). More specifically, 1991 census data were used for 2000–2004 draftees, while 1996 census data were used for 2005–2014 draftees. In order to test for heterogeneity in NHL talent production, the population size of all Canadian cities registered in the 1991 and 1996 census data (i.e., productive and unproductive cities) were used in this study.

All Canadian cities were then categorized into population size subdivisions. The following categories were used as they correspond to the Canadian census population categories, which would facilitate comparison to Canadian general population data, as well as to previous literature on the community size effect in similar athlete populations (Baker and Logan, 2007; Baker et al., 2014; Wattie et al., 2018): <2,500; 2,500–4,999; 5,000–9,999; 10,000–29,999; 30,000–99,000; 100,000–249,999; 250,000–499,999; 500,000–999,999; >1,000,000. Given that cities within certain categories, especially larger ones, may vary substantially in population size, the number of players produced per 1,000 residents (i.e., yield) was introduced in this study as an athlete-development metric that has not been previously used in community size effect research. Yield values will account for potentially large differences in population size that may exist within categories, and will allow for comparison in athlete production between cities in different categories while accounting for population size. This project was approved by the University of Ontario Institute of Technology Research Ethics Board.

### Analysis

Inferential statistics were not used in this manuscript for three reasons. First, data collected for this research consists of a complete population of all Canadian hockey players drafted into the NHL during a 15-year period, and therefore is not a sample collected from a larger population to which its statistics can be inferred. The common use of inferential statistics in sport has been recently criticized by Gibbs et al. (2015) who argued that inferring findings in sport research may be misleading as team rosters, league statistics, or general information pertaining to athletes constitute their own independent populations and should not be mistaken for samples. Second, much of the country’s census structure has changed since the players in this dataset were born. For example, the city of Winnipeg’s population has changed by 300,000 people, Toronto has seen an increase in population size by over two million residents, and the total population of Canada has grown from 28,713,070 in 1991 to 36,626,083 people in 2016. Given the significant census changes that have occurred over the past 15 years, and taking into account possible mergers and segregations of cities, it may not be appropriate to infer findings from the 1990s to the current state of the country. Third, the dataset used for this study includes 5228 Canadian cities, 411 of which have produced at least one NHL draftee over the period of the study; therefore, conducting comparisons for every city that has produced draftees would have increased the Type 1 error rate to a point what would have obfuscated the trends of the study, not to mention that carrying out such a large number of comparisons would have been cumbersome.

In lieu of inferential statistics, the current study provides a descriptive exploration of heterogeneity of community size effects within population size categories. For the first step of our purpose, the number of productive cities was divided by the total number of cities in each category to determine the percentage of productive cities within each population size category. Considering the fact that population sizes of cities tend to fluctuate over time, moving cities higher or lower on the categorical scale, median population sizes were drawn for each city from the 1991 and 1996 census data and were then used to place cities in their respective population size categories. Due to regional mergers and amalgamations, population size data was available in one census year for some cities, in which case the provided population size was used and not the median value. However, these cities accounted for only 7% of the total dataset. For the second step of the purpose (i.e., examining the variation in NHL talent production between productive cities), the population mean (μ), population standard deviation (σ), as well as minimum and maximum values (range) of NHL draftees were calculated for each population size category in every provincial region. Moreover, the yields of cities were calculated by dividing the number of NHL draftees each city had produced between 2000–2014 over its population size, then multiplying that figure by 1,000 (to generate yield per capita).

## Results

**FIGURE 1:**
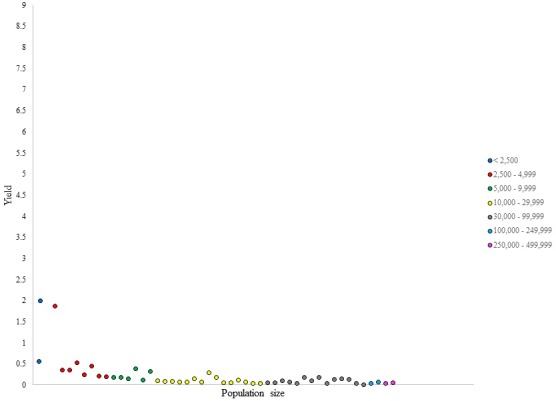
Number of NHL draftees per 1,000 residents (i.e., yield) per city in British Columbia (cities ranked in ascending population size).

**FIGURE 2:**
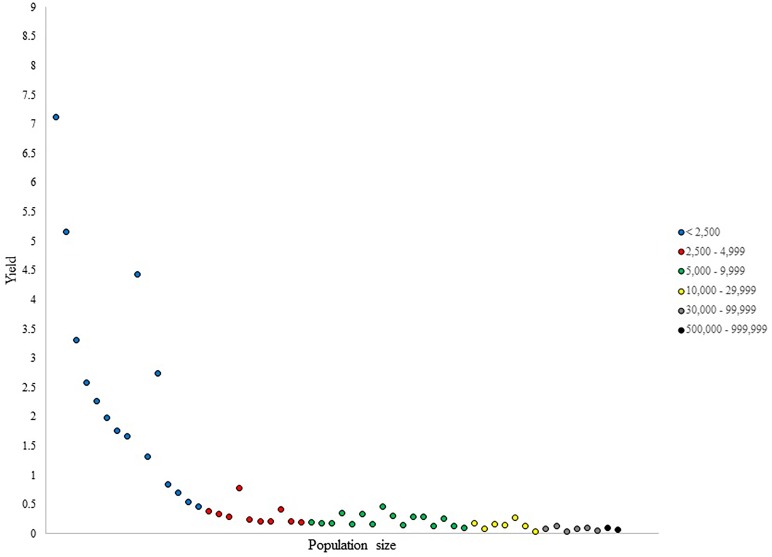
Number of NHL draftees per 1,000 residents (i.e., yield) per city in Alberta (cities ranked in ascending population size).

**FIGURE 3:**
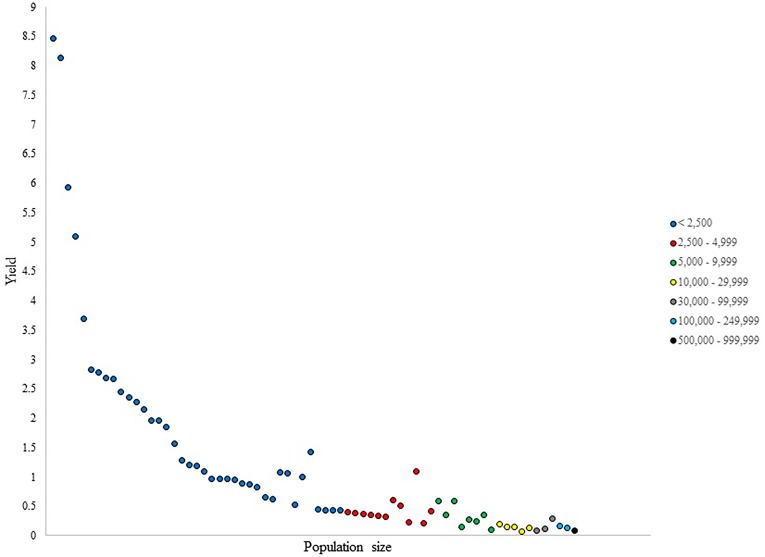
Number of NHL draftees per 1,000 residents (i.e., yield) per city in Saskatchewan and Manitoba.

**FIGURE 4:**
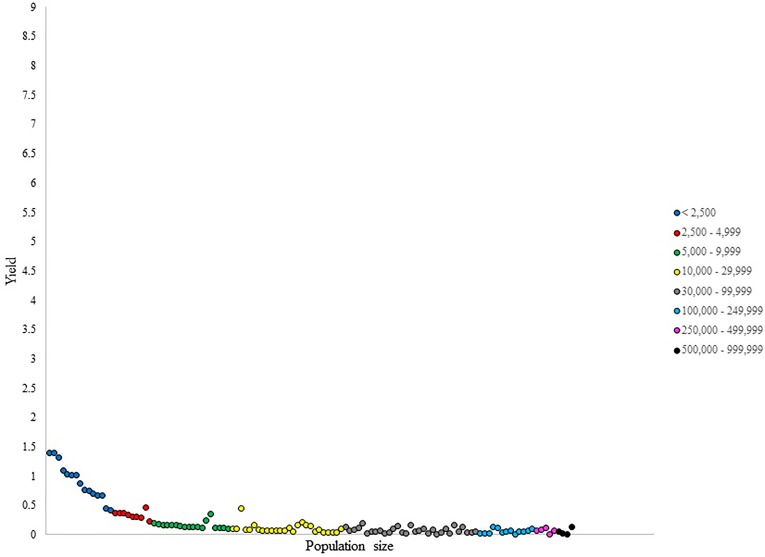
Number of NHL draftees per 1,000 residents (i.e., yield) per city in Ontario (cities ranked in ascending population size).

**FIGURE 5:**
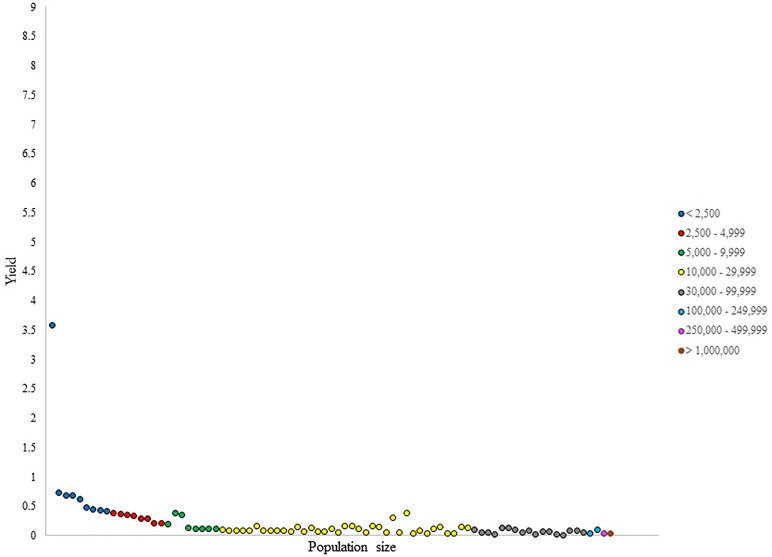
Number of NHL draftees per 1,000 residents (i.e., yield) per city in Quebec (cities ranked in ascending population size).

**FIGURE 6:**
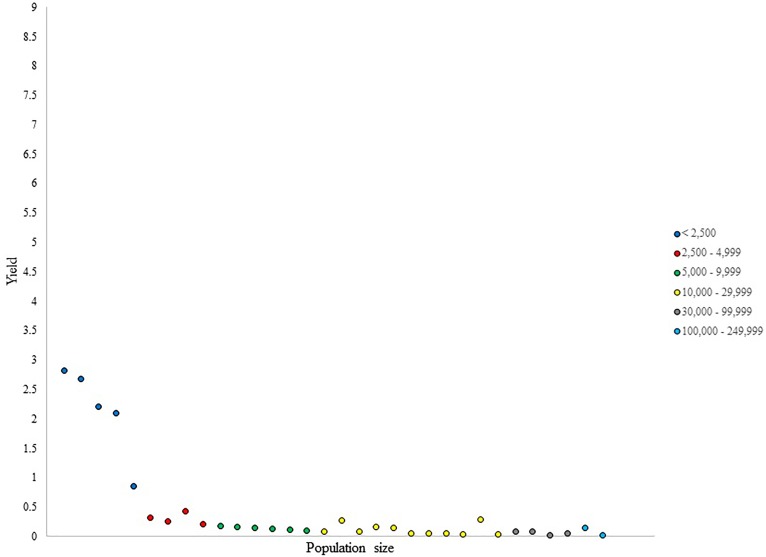
Number of NHL draftees per 1,000 residents (i.e., yield) per city in Atlantic Provinces (cities ranked in ascending population size).

Due to the considerable variation in populations, density, etc. between regions of Canada, results are presented for each provincial region separately, moving from west to east in Canada. Variations in production between and within population size categories are presented in Table 1. This table includes the total number of cities, the number and proportion of productive cities, as well as the total number of draftees produced in each population size category in all provincial regions. Table 2 displays the average, standard deviation, and range values of NHL draftees produced for each population size category in all provincial regions. Figures 1–6 outline the production of players per 1,000 residents (i.e., yield) for each city within each population size category, in each provincial region of Canada. Cities in these graphs are ranked from left to right from the lowest to the highest population size, and are patterned according to the category they fall in.

**Table 1:** Variability in the production of NHL draftees within and between population size categories in each provincial region.

		<2,500	2,500–4,999	5,000–9,999	10,000–29,999	30,000–99,999	100,000–249,999	250,000–499,999	500,000–999,999	>1M
**No. of cities**	BC	298	43	40	42	18	2	2	NA	NA
	AB	295	56	51	21	8	NA	NA	2	NA
	SK and MB	1077	42	20	6	3	2	NA	1	NA
	ON	513	144	95	63	38	15	5	4	NA
	QC	1074	173	77	83	34	2	1	NA	1
	ATL	690	90	54	27	7	2	NA	NA	NA
**No. of productive cities (%)**	BC	2 (0.67%)	8 (18.6%)	6 (15%)	15 (35.7%)	14 (77.8%)	2 (100%)	2 (100%)	NA	NA
	AB	15 (5.1%)	10 (17.9%)	16 (31.4%)	7 (33.3%)	6 (75%)	NA	NA	2 (100%)	NA
	SK and MB	39 (3.6%)	12 (28.6%)	8 (40%)	5 (83.3%)	3 (100%)	2 (100%)	NA	1 (100%)	NA
	ON	15 (2.9%)	9 (6.25%)	18 (18.9%)	26 (41.3%)	31 (81.6%)	13 (86.7%)	5 (100%)	4 (100%)	NA
	QC	9 (0.84%)	8 (4.6%)	8 (10.4%)	37 (44.6%)	17 (50%)	2 (100%)	1 (100%)	NA	1 (100%)
	ATL	5 (0.72%)	4 (4.4%)	6 (11.1%)	11 (40.7%)	4 (57.1%)	2 (100%)	NA	NA	NA
**Total no. of draftees**	BC	2	14	10	25	87	18	36	NA	NA
	AB	19	13	24	14	26	NA	NA	122	NA
	SK and MB	44	19	18	9	18	54	NA	54	NA
	ON	15	10	21	44	134	107	103	127	NA
	QC	10	8	10	74	56	23	14	NA	46
	ATL	6	5	6	21	17	19	NA	NA	NA

*BC, British Columbia; AB, Alberta; SK and MB, Saskatchewan and Manitoba; ON, Ontario; QC, Quebec; ATL, Atlantic Province; NA, not applicable.*

**Table 2:** Population average, population standard deviation, and range values of NHL draftees per city across population size categories in each provincial region.

Population size category	BC μ (±σ)	AB μ (±σ)	SK and MB μ (±σ)	ON μ (±σ)	QC μ (±σ)	ATL μ (±σ)
<2,500	0.01 (±0.08) (0–1)	0.06 (±0.32) (0–3)	0.04 (±0.22) (0–3)	0.03 (±0.17) (0–1)	0.01 (±0.11) (0–2)	0.01 (±0.11) (0–2)
2,500–4,999	0.33 (±0.89) (0–5)	0.23 (±0.57) (0–3)	0.45 (±0.94) (0–5)	0.07 (±0.28) (0–2)	0.05 (±0.21) (0–1)	0.06 (±0.27) (0–2)
5,000–9,999	0.25 (±0.70) (0–3)	0.47 (±0.78) (0–2)	0.90 (±1.29) (0–4)	0.22 (±0.51) (0–3)	0.13 (±0.41) (0–2)	0.11 (±0.32) (0–1)
10,000–29,999	0.60 (±1.06) (0–5)	0.67 (±1.11) (0–4)	1.5 (±0.84) (0–2)	0.7 (±1.10) (0–5)	0.9 (±1.45) (0–8)	0.77 (±1.48) (0–7)
30,000–99,999	4.83 (±4.62) (0–13)	3.25 (±2.43) (0–6)	6 (±4.36) (3–11)	3.53 (±3.27) (0–13)	1.65 (±2.09) (0–6)	2.43 (±2.06) (0–6)
100,000–249,999	9 (±4.25) (6–12)	NA	27 (±4.24) (24–30)	7.13 (±6.14) (0–19)	11.5 (±9.19) (5–18)	9.5 (±7.78) (2–17)
250,000–499,999	18 (±12.73) (9–27)	NA	NA	20.6 (±12.20) (4–37)	NA	NA
500,000–999,999	NA	61 (±5.66) (57–65)	NA	31.75 (±38.19) (3–87)	NA	NA
>1,000,000	NA	NA	NA	NA	NA	NA

*BC, British Columbia; AB, Alberta; SK and MB, Saskatchewan and Manitoba; ON, Ontario; QC, Quebec; ATL, Atlantic Provinces; NA, not applicable; μ, population average; σ, population standard deviation.*

### British Columbia

The proportion of productive cities seemed to generally increase as population size increased, with the exception of the two largest categories (500,000–999,999 and >1,000,000), which did not exist in British Columbia (see Table 1). The category of 30,000–99,999 produced the highest number of NHL draftees at 87; however, some cities in this category (22.2%, e.g., Kamloops) did not produce any prospects, while the city of Victoria produced 13. The categories of 100,000–249,999 and 250,000–499,999 were the only categories in which all cities produced players, and they presented the highest μ values of NHL prospects produced per city at 9 and 18, respectively. However, their respective σ were 4.25 and 12.73, and the difference in the number of prospects produced between the least and most productive cities was twofold in the 100,000–249,999 category, and threefold in the 250,000–499,999 category.

For only the productive regions, 22 out of 49 of those cities produced only one player. The number of prospects per city generally increased with population size, with Vancouver producing the highest number of draftees at 27. Despite this general trend, six smaller cities produced more prospects than the second most populous city in British Columbia. Overall, there was less variation observed in the smallest four categories than the biggest three categories.

For the yield (i.e., the production of NHL draftee per capita), the data presented in Figure 1 revealed an expected decrease with increasing population size, given the high number of cities that have produced only one NHL prospect in the smaller categories. Nevertheless, outliers were identified for their ability to generate prospects per capita at a higher capacity than other cities within their respective categories. For instance, one city presented a yield of 1.8, which was considerably larger than the values presented by other cities in its category, which ranged from 0.2 to 0.5. Similarly, two outliers were observed in the category of 5,000–9,999 with yield values of 0.38 and 0.32, respectively. The distribution of yield values within population size categories was not uniform, even in larger categories, where the difference in population size between the least and the most populous city can be quite large.

### Alberta

The proportion of productive cities in Alberta, outlined in Table 1, increased with population size; however, so did the standard deviation of players produced, indicating increasing variability within these categories. The largest population size category in this province (500,000–999,999) produced the highest μ of players, and the number of players per city was quite comparable at 57 and 65 prospects. As shown by the range values in Table 2, some variability was observed in the smaller categories. Most notably, the category of 30,000–99,999, which included eight cities and a total production of 26 draftees. Similarly, in the remaining categories, some cities were capable of producing two, three, or four prospects while other cities within their categories did not produce any.

When excluding the unproductive cities, a relatively low level of variability was observed within population size categories in Alberta, as the range values between the least and the most productive cities were 2, 2, 2, 3, 4, and 8 in the <2,500; 2,500–4,999; 5,000–9,999; 10,000–29,999; 30,000–99,999; and 500,000–999,999 categories, respectively.

The <2,500 population size category in Alberta, displayed in Figure 2, presented a large variability in yield values. This is due to some remarkably small towns producing as many draftees as larger cities in this category. Two outliers in this category were seen with yield values of 4.4 and 2.7. The 2,500–4,999 category was somewhat uniform in its distribution of yield values within its cities; however, two cities stood out in their production of draftees per capita. Although no clear outliers were observed in the 5,000–9,999 category, the distribution of yield values in the 5,000–9,999 category were non-uniform, with the same pattern of results observed in the 10,000–29,999 category. Conversely, the two largest categories in this province did not display much heterogeneity in their per capita production.

### Saskatchewan and Manitoba

The provinces of Saskatchewan and Manitoba included 1,077 regions in the <2,500 category, only 39 of which produced an NHL draftee. Despite the low μ of NHL prospects in that category, some regions produced three NHL prospects, while other, similarly sized, areas did not produce any. The total number of players produced in the 30,000–99,999 category was 18; however, 11 of these prospects were from a single city. Similar to Alberta, the category of 250,000–499,999 did not exist in Saskatchewan and Manitoba.

Despite the low proportion of productive cities in the category of <2,500, and the low average of NHL draftees per city, one city in that category produced more prospects than any city in the 10,000–29,999 category, and as many prospects as Moose Jaw, a city with a population size of over 33,000 people. Moreover, the category of 2,500–4,999 had 12 productive cities, eight of which only produced one NHL draftee, but one city had produced five. The remaining categories in this provincial region did not reflect as much variation within its cities, with the exception of the 30,000–99,999 category, where one region produced 11 prospects, compared to four and three prospects produced by the rest.

Figure 3 shows a general decrease in yield values as population size increased. The smallest category in Saskatchewan and Manitoba did not present uniform yield values within its cities, as there were two towns in this category with yield values of over 8, as both cities produced one NHL draftee despite their small population sizes of 118 and 123. The category of 2,500–4,999 did not present uniformity in yield values either, as two clear outliers in their per capita production were observed. Similarly, in the 5,000–9,999 category, yield values were heterogeneous, with two cities producing more players per capita than similarly sized cities.

### Ontario

The smallest three categories in Ontario did not reveal much variability as indicated by the range values; however, there was considerable variability in the larger categories. For instance, some cities in the 30,000–99,999 category did not produce any draftees, while other cities produced 9, 11, and 13 prospects. The category of 100,000–249,999 had a high μ of 7.13 NHL draftees per city, yet two cities in that category were not productive at all, showing that statistics such as the mean or odds can be misleading when generalized across all regions within a category. Moreover, both μ and σ values in the 250,000–499,999 and 500,000–999,999 categories indicated substantial heterogeneity in NHL talent production.

There was no observed variability between productive cities in the smallest category, as all 15 cities had each produced one draftee, and little variability overall in the smallest four categories in general. However, in the category of 100,000–249,999 people, the production of one city far exceeded certain cities in that category with 19 prospects. Similarly, in the 250,000–499,999 category, Ottawa produced 11 more players than the second most productive city in that category, and 33 more than the least productive city. In the largest category, Toronto’s production of prospects was substantially superior to the other cities. However, it is important to note that Mississauga, Scarborough, and North York are parts of the Greater Toronto Area (GTA); therefore, it may be that draftees were born in Toronto hospitals but developed in other regions of the GTA, or vice versa. Nevertheless, census statistics as well as place of birth data do reflect heterogeneity in NHL talent production in that category.

Yield values in Ontario, displayed in Figure 4, showed a decrease with increasing population size in the smallest four categories, with five outliers being observed. The larger categories displayed no uniformity in NHL talent production per capita within their cities, suggesting no relationship between population size and the number of draftees produced per capita.

### Quebec

The province of Quebec included 1,074 cities in the <2,500 category, only nine of which produced players, with little variability shown within this category and the two categories above. The category of 10,000–29,999 contributed more to Quebec’s total production of prospects than any other category, with 74 NHL draftees. However, over half of the cities in that category did not produce any players. The 30,000–99,999 category had the second highest production of NHL prospects, yet only 17 out of 34 cities in that category managed to produce at least one draftee. Moreover, the 100,000–249,999 category generated 23 prospects in total, 18 of them originated from a single region.

Given the relatively small range values in the smallest three categories, little variability was observed between the productive cities of those sizes. The larger categories, however, displayed a higher level of variability between its productive cities. Although 21 out of 37 productive cities in the 10,000–29,999 category only produced one draftee, two cities were clear outliers in that group; each producing six and eight draftees. Unlike the 10,000–29,999 category, the outliers in the 30,000–99,999 category were cities that only produced one player as the majority of cities of that population size produced three or more players.

Figure 5 presents the yield in Quebec revealing a clear outlier in the <2,500 category which had a yield of 3.6, compared to other cities in that category which did not exceed the value of 0.7. Little variability was shown in the 2,500–4,999 and 5,000–9,999 categories; however, two regions stood out in their per capita production relative to other cities within their category. The remaining categories did not display uniformity in yield, suggesting that cities within them were not equal in their per capita production of draftees.

### Atlantic Provinces

Similar to Quebec, the three smallest population size categories did not present much variability in NHL prospect production, but the larger categories did. Sixteen cities in the 10,000–29,999 category did not produce any draftees, but one city produced seven, which was the second highest production in these provinces. Similarly, in the 30,000–99,999 category, three cities did not generate any NHL draftees, one area generated the third highest number of prospects in the Atlantic Provinces (*n* = 6).

As for the 30,000–99,999 category, one region produced only two players while other cities in this category were more productive; producing between four and six draftees. In the 100,000–249,999 category, Halifax produced 8.5 times as many players as Cape Breton, despite the difference in their population sizes being about 500 people.

As for the production of NHL draftees per capita, yield values were heterogeneous in the <2,500 category, as two cities had each produced one draftee with population sizes of only 355 and 373, respectively, giving them much higher yield values than the remaining cities in this category. Bigger categories displayed more uniformity in per capita production. Although, certain outliers stood out from other cities in their categories.

## Discussion

The purpose of this project was to examine the heterogeneity in community size effects within and between population size categories across different provincial regions of Canada. This was done by examining the variation in the proportion of productive cities in each category, as well as the number of players produced by each city within categories. In addition, given that the difference in population size within certain categories can be large, the number of players produced per 1,000 residents was introduced as a metric of NHL talent development to help compare the production of draftees within population size categories. Results from this study suggest that descriptive statistics of community size effects may not be generalizable, given that the categories with the highest total of draftees and the highest average of draftees produced were not consistent between provinces. Moreover, when examining such categories within each province individually, substantial variation was revealed. In addition to variability in the number of players produced, intra-categorical yield values showed similar heterogeneity and non-uniformity of player production per capita within both highly productive and less productive categories. Therefore, not only does variability in Canadian NHL talent production exist between similarly sized cities, it exists in various degrees in different provinces, suggesting a single homogenous community size effect may not exist for NHL athletes in Canada.

When comparing NHL talent production within and between population size categories across provincial regions, results showed inconsistencies with some of the findings of previous community size effect studies in Canadian NHL talent. Côté et al. (2006), for example, concluded that the optimal population size to develop Canadian NHL players was between 1,000 and 500,000 residents, as players from towns with less than 1,000 or greater than 500,000 residents were underrepresented. However, results from the current study reveal significant variability in NHL talent production exists within population size categories that fall within that range. For instance, 100 cities in British Columbia, 97 cities in Alberta, 43 cities in Saskatchewan and Manitoba, 258 cities in Ontario, 297 cities in Quebec, and 161 cities in the Atlantic Provinces with population sizes between 2,500 and 500,000 did not produce any NHL draftees from 2000–2014. Similarly in Alberta, Daysland had a population size of 676 inhabitants, yet it had produced as many draftees as Fort McMurray, which had a population size of 34,700. Daysland also managed to produce more draftees than 15 of the productive cities in 5,000–9,999 category, and six out of seven productive cities in the 10,000–29,999 category. In Quebec, Sainte-Agathe produced two NHL draftees with only 558 inhabitants, which is more than what 21 cities in the 30,000–99,999 managed to produce.

Baker and Logan (2007) identified the categories of 100,000–249,999 and 500,000–999,999 to be the most advantageous in terms of developing NHL draftees. However, there were no cities with 500,000–999,999 inhabitants in British Columbia, Saskatchewan, Quebec, or any of the Atlantic Provinces, meaning that the findings for this category cannot be generalized across seven out of 10 Canadian provinces. Although Ontario had four cities in that category, their production of NHL draftees varied markedly. As for the 100,000–249,999 category, there were no cities of that size in Alberta. Even in provinces where that category did exist, heterogeneity was observed within cities of that size. For instance, in British Columbia, Richmond and Burnaby fell into that category, yet Burnaby produced twice the number of prospects as Richmond; moreover, six smaller cities produced more draftees than Richmond. Similarly, in the Atlantic Provinces, Halifax produced 17 prospects compared to only two by Cape Breton, with 10 smaller cities matching or exceeding Cape Breton’s production.

Just as previous research indicated that aggregating athlete production data on a national level obscures substantial variation between provincial regions (Wattie et al., 2018), results of the current study indicate similar aggregation of data through the use of population size categories masks equally meaningful variation in NHL talent development between Canadian cities. In addition to masking heterogeneity in athlete production, the use of population size categories masks large differences in population size as well. For instance, the difference in population size between two cities in the 500,000–999,999 category can be close to half a million residents. Consequently, this puts the accuracy and generalizability of this effect into question.

Several sociocultural factors may have contributed to intra-categorical variation in NHL talent development observed in this study. For instance, given that specific sports are valued differently across cultures, and that hockey is predominantly participated in by players of Caucasian descent, one of such factors may be the ethnic diversity of certain cities. For example, in the 30,000–99,999 category in Ontario, Ajax and Sault Ste. Marie produced substantially different numbers of draftees with 4 and 13, respectively. The 2011 National Household Survey from both cities revealed that visible minorities form 45.8% of Ajax residents, and only 0.03% in Sault Ste. Marie (Statistics Canada, 2013).

In addition to ethnicity, household income may be a significant socioeconomic contributor to athlete development especially in a costly sport such as hockey, and may be one of the factors causing intra-categorical variability. A survey conducted by Hockey Canada indicates that the 2011–2012 minor hockey season cost parents approximately $3000 per child (Mirtle, 2013), with some “A” or “AA” level leagues such as the Greater Toronto Hockey League charging over $5000 per season (Pom, 2014). Moreover, the cost of private hockey academies ranges from $35,000 to over $50,000 (Cole, 2015). Such high costs of participation may deter a sizable portion of Canadians from enrolling their children in organized hockey past a certain level of competition, thus eliminating their chances of developing into NHL draftees.

Although the current research extends the existing body of literature on community size effects, a notable limitation of this study is that the use of 1991 and 1996 census data may not provide an exact measurement of population size for the athletes’ places of birth during their developmental years. However, Canadian census data are updated periodically every 5 years, meaning that depicting the population size of a region during any chosen year is not exact. Similarly, place of birth data collected from the NHL website may not always be an accurate representation of the place of development for some of the players in this data set, which is a common limitation of the majority of community size effect studies (Finnegan et al., 2017). For example, Toronto produced 87 NHL draftees in 2000–2014; however, it is not clear whether some of those players grew up in the city of Toronto during their developmental years or whether they were born in a Toronto hospital but spent their childhood years in one of many Greater Toronto Area suburbs. Moreover, while findings from this research provide novel information on the importance of early developmental environments, they do not provide any indication of the factors driving the variation observed. For example, two different cities could have a population size of 50,000 residents, yet one city may have a much smaller surface area, thus increasing the number of residents per square kilometer. Differences in population density may help explain the variation in NHL talent production within population size categories; Rossing et al. (2016) found that regions of low population density (<1,000 residents/km^2^) produced more elite handball players in the Denmark, while regions of high population densities (>1,000 residents/km^2^) produced more elite football players. Recent literature has studied the influence of population density on athlete development in youth Danish handball and football players (Rossing et al., 2018) and in elite male and female Portuguese handball players (Hancock et al., 2017). However, whether or not population density has an effect on the development of NHL draftees may be an important area for future research.

Another suggested mechanism of the community size effect is the cultural and familial support surrounding athletes. Small-medium sized regions are thought to be more conducive to a positive integration of family, school and community support than large urban centers (Fraser-Thomas et al., 2010; Surya et al., 2012; Balish and Côté, 2014). Moreover, small communities are more likely to take pride in their local sport teams and promote a culture that emphasizes the importance of sport participation (Bale, 2003). This may also motivate more local parents and adults to be involved in youth sport as coaches, referees, league conveners, and many other roles. However, factors such as pride and loyalty are difficult to measure, and in turn, difficult to use as an explanation for a tangible outcome such as the number of players produced. Moreover, they may not be generalizable to all small-medium sized towns, as many of those regions may not have local sport teams to follow and take pride in. There are three amateur hockey leagues in Canada that make up The Canadian Hockey League (CHL): the Western Hockey League (WHL), the Ontario Hockey League (OHL), and the Quebec Major Junior Hockey League (QMJHL), each allocated certain regions of Canada in which they are allowed to scout and draft players typically at 15–16 years of age. The CHL is renowned not only as the highest level of amateur hockey in Canada, but as one of the best developmental amateur leagues for hockey players prior to the NHL draft. Growing up in a city that has a CHL team, or close to it, could provide communities with an opportunity to culturally identify by a local team, and take pride in following. Moreover, it may provide minor hockey players with opportunities to be seen by CHL scouts, which could increase their chances of being drafted into the CHL, thus exposing them to NHL scouting later on. Therefore, the proximity to CHL teams may help shed more light on why variation exists within population size categories. Whether such mechanisms are present across all cities within advantageous population size categories, and absent in disadvantageous categories, may provide insights on the observed variation in the current study.

## Conclusion

Results from this study reveal heterogeneity in the production of NHL prospects between and within population size categories in Canadian provinces. These findings suggest population size is not an accurate or consistent predictor of NHL talent production. Future research should consider the specific environmental and developmental characteristics of certain cities that may make them more advantageous in developing NHL prospects than others. Heterogeneity within population size categories also suggest that the hypothesized mechanisms of the community size effect are indeed plausible, but that they may not apply uniformly across cities of similar sizes, as sharing the same population size as another city does not equate to possessing similar characteristics that foster athlete development. A greater understanding of the specific environmental and developmental characteristics of cities may help us understand this heterogeneity in NHL talent production within and between population size categories, and extend our knowledge about the geography of athlete development.

## Author Contributions

LF was the primary author, conducted the analysis on previously obtained public data, and wrote the manuscript with the help and guidance of co-authors. JS contributed to formulate the structure of this project, developed the rationale, and edited the manuscript. JB contributed greatly to co-develop the method of statistical analysis, structured the layout of the results and discussion. NW contributed to develop the methods along with JB, and co-wrote and edited the manuscript.

## Conflict of Interest Statement

The authors declare that the research was conducted in the absence of any commercial or financial relationships that could be construed as a potential conflict of interest.

## Footnotes

1 NHL.com

2 For a visual representation of Canada’s provinces, refer to the following provincial map: www.map-of-canada.org/map-of-canada-724.jpg.
